# Towards Next-Generation Sustainable Composites Made of Recycled Rubber, Cenospheres, and Biobinder

**DOI:** 10.3390/polym13040574

**Published:** 2021-02-14

**Authors:** Kristine Irtiseva, Vjaceslavs Lapkovskis, Viktors Mironovs, Jurijs Ozolins, Vijay Kumar Thakur, Gaurav Goel, Janis Baronins, Andrei Shishkin

**Affiliations:** 1 Rudolfs Cimdins Riga Biomaterials Innovations and Development Centre of RTU, Institute of General Chemical Engineering, Faculty of Materials Science and Applied Chemistry, Riga Technical University, Pulka 3, Riga LV-1007, Latvia; kristine.irtiseva@rtu.lv (K.I.); jurijs.ozolins@rtu.lv (J.O.); andrejs.siskins@rtu.lv (A.S.); 2 Scientific Laboratory of Powder Materials & Institute of Aeronautics, 6B Kipsalas Str., Faculty of Mechanical Engineering, Riga Technical University, Riga LV-1048, Latvia; viktors.mironovs@rtu.lv; 3 Biorefining and Advanced Materials Research Center, Scotland’s Rural College (SRUC), Kings Buildings, West Mains Road, Edinburgh EH9 3JG, UK; vijay.thakur@sruc.ac.uk; 4 School of Engineering, London South Bank University, 103 Borough Road, London SE 10AA, UK; goelg@lsbu.ac.uk; 5 Maritime Transport Department, Latvian Maritime Academy, 12, Flotes Str., k-1, Riga LV-1016, Latvia; jbaronins@gmail.com

**Keywords:** sustainable composites, crumb rubber, devulcanised crumb rubber, cenosphere, peat, biocomposite, hybrid material, bio-binder, oil absorption

## Abstract

The utilisation of industrial residual products to develop new value-added materials and reduce their environmental footprint is one of the critical challenges of science and industry. Development of new multifunctional and bio-based composite materials is an excellent opportunity for the effective utilisation of residual industrial products and a right step in the Green Deal’s direction as approved by the European Commission. Keeping the various issues in mind, we describe the manufacturing and characterisation of the three-component bio-based composites in this work. The key components are a bio-based binder made of peat, devulcanised crumb rubber (DCR) from used tyres, and part of the fly ash, i.e., the cenosphere (CS). The three-phase composites were prepared in the form of a block to investigate their mechanical properties and density, and in the form of granules for the determination of the sorption of water and oil products. We also investigated the properties’ dependence on the DCR and CS fraction. It was found that the maximum compression strength (in block form) observed for the composition without CS and DCR addition was 79.3 MPa, while the second-highest value of compression strength was 11.2 MPa for the composition with 27.3 wt.% of CS. For compositions with a bio-binder content from 17.4 to 55.8 wt.%, and with DCR contents ranging from 11.0 to 62.0 wt.%, the compressive strength was in the range from 1.1 to 2.0 MPa. Liquid-sorption analysis (water and diesel) showed that the maximum saturation of liquids, in both cases, was set after 35 min and ranged from 1.05 to 1.4 g·g ^−1^ for water, and 0.77 to 1.25 g·g^−1^ for diesel. It was observed that 90% of the maximum saturation with diesel fuel came after 10 min and for water after 35 min.

## 1. Introduction

In the modern world, human civilisation is suffering from many challenges, such as an extensive increase in the generated waste stream by plastic-material pollution and, at the same time, lacking new efficient (lightweight, recyclable, or decomposable, made of biosourced or recycled raw materials) materials. 

Among various waste materials, cenosphere (CS) is a low-density (0.25–0.55 g·cm^−3^) [1], inert, nontoxic, nonflammable, powder-like material which is a part of fly ash. Cenospheres have emerged as beneficial additives for several applications with their hollow structure and lightweight properties. These materials are primarily derived from coal fly ash, which is a significant pollutant all over the world. Thus, the application of cenospheres in composite-materials design contributes to a circular economy concept. Cenospheres have been chosen as a component in previous works for their specific properties such as low bulk density, high thermal resistance, good workability, and high strength [1]. Its addition to composite materials helps make the material lightweight and improves absorption and acoustic properties [2,3,4,5]. They may also impose some adverse effects on physical properties such as reduced compressive strength and increased porosity [2,6]. A decision on the trade-off between these various factors, such as lightweight, compressive strength, cost-effectiveness, etc., is essential in developing the material with the desired properties.

Every year, millions of tyres are discarded across the world, representing a severe threat to the ecology along with the fly ash. By the year 2030, up to 5000 million tyres could be discarded regularly [7]. Discarded tyres often lead to “black pollution” because they have a long life, are non-biodegradable, and pose a potential threat to the environment [8]. The traditional waste-tyres management methods have been stockpiling, illegal dumping, or landfilling, all of which are short-term solutions. The growing amount of scrap-tyre waste has created a tremendous amount of waste being dumped which is not biodegradable. As Europe is taking the lead in recycling efforts, their use as fuel in the steel industry, cement industry, and incineration facilities is being promoted [9]. In the past, some efforts have been made by developing composite from fly ash and waste-tyre powder [10], and geopolymer from fly ash and waste tyre [11]. Alternatively, waste tyres are also being used to create running tracks, playgrounds, artificial turf, railways, and in road building [12]. The utilisation of crumb rubber is also gaining attention by incorporation into concrete and rubberised asphalt [13]. There is currently more drive in developing sustainable biocomposite materials using fly ash and tyre waste involving other bio-based materials. A biocomposite is a category of biocompatible and environmentally friendly composites that are biopolymers consisting of natural fibres. Biocomposites are composed of a wide range of organic and inorganic components such as natural and synthetic polymers, polysaccharides, proteins, sugars, ceramics, metal particles, and hydrocarbon nanoparticles. Biocomposites come in various forms such as films, membranes, coatings, fibres, and foams. There are several examples of using peat/sapropel binders, such as sapropel concrete, birch-wood fibre, sanding dust, and hemp shives, for composite materials [14,15]. These materials may be in the form of blocks or pellets. Literature studies have shown the possibility of using sapropel/peat as a raw material in ecological construction. They can be considered promising materials for building materials and designing products [16,17].

Extensive research has been carried out to improve materials’ mechanical properties and functionality and develop environmentally friendly composite materials [18,19,20]. Some related attempts on the recent development of composites with improved performance have been reported [21,22,23,24]. The use of bio-binders is essential for developing these biocomposites [25]. Bio-binders, also called biopolymers, are compounds derived from natural resources and are composed of monomer units covalently linked to form larger structures [26,27]. An example of a bio-binder is natural fibres. Natural binders differ in melt flow rate, impact properties, hardness, vapour permeability, and friction and decomposition coefficient. The water absorption of the bio-binder will also vary depending on the chemical composition of the bio-binder’s processing conditions [28]. The production of bio-based polymers using renewable materials has gained significant attention in recent decades because of the United Nation’s Sustainable Development Goals’ achievement. Latvia and the Baltic region are extraordinarily rich in natural peat. One aim of the work is to investigate the possibility of a new application of natural peat as a bio-binder for hybrid composite materials. 

Through this research, the authors introduce new biocomposite materials made of two recycled materials: a cenosphere and a devulcanised crumb rubber, and a bio-sourced binder made of natural peat. For the first time, this study proposes the use of crumb rubber along with cenosphere and a natural binder, peat, in developing a composite material. These solutions are in line with the United Nations sustainable development goals by fostering the conversion of waste materials into value-added products.

We describe here the utilisation of devulcanised crumb rubber (DCR), homogenised peat (HP), and cenospheres (CS) for composite-material development with a bio-binder. This research is aimed to answer the question about what effect the main component DCRHP-CS content has on the composite material properties such as density, mechanical properties, and the absorption of water and oil products.

## 2. Materials and Methods 

### 2.1. Raw Materials and Compositions

For the manufacturing of sustainable composite material in two forms, blocks and granules, a bio-binder made of HP, DCR, and CS was used. Three general compositions with a CS content of 0.0, 5.0, and 10.0 wt.% in a wet mixture were used. For each composition, DCR amounts of 0.0, 5.0, 10.0, 15.0, 20.0, and 30.0 wt.% were chosen. Samples designations and composition of the studied materials in blocks and granules are presented in Table 1 and Table 2. For the production of the specimen, the wt.% of HP in wet condition (suspension with water content 85 wt.%) was used, but the real DCR, CS, and HP content after drying is also represented in Table 1 and Table 2 for an understanding of the entire composition of the studied materials. 

**Figure 1:**
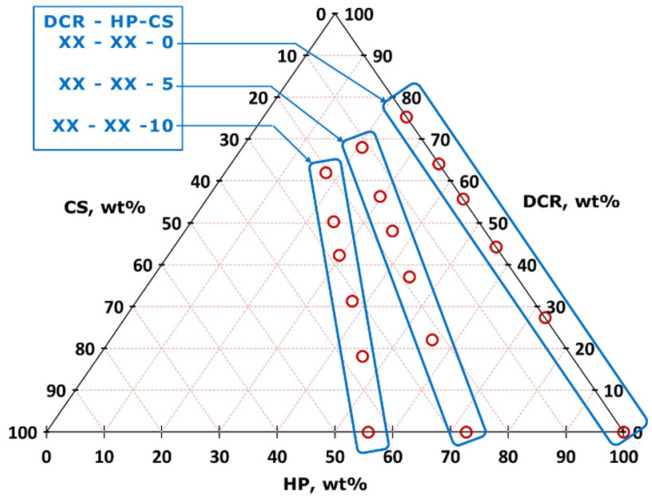
Ternary diagram of the dried composed material composition by wt.%. Three groups of composition classified by CS content (XX–XX–0, XX–XX–5, and XX–XX–10) are indicated.

For a better understanding, all the studied recipes are represented in a ternary composition diagram in Figure 1. Three groups of composition, classified by a cenosphere (CS) content in the wet composition of 0, 5, and 10 wt.% correspond to the sample series XX–XX–0, XX–XX–5, and XX–XX–10, respectively.

**Figure 2:**
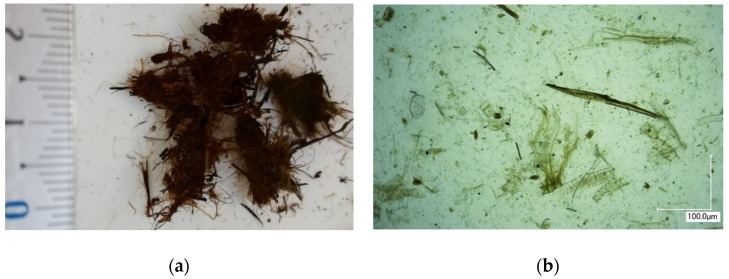
Peat agglomerates (**a**) before and peat particles (**b**) after treatment by high-speed multidisc mixer–disperser (HSMD).

Natural peat (deposition *Keizerpurvs*, Cesis, Latvia) was preliminarily processed through a hydrocavitation process for use as a bio-based binder. The raw peat (humidity 65–70%) was mixed with water and processed in a high-speed multidisc mixer–disperser (HSMD) with cavitation effect for obtaining the homogeneous water–peat slurry with dry matter contents of 15 ± 1 wt.%. Raw peat agglomerates before, and peat particles (extracted from the suspension) after treatment by HSMD, are shown in Figure 2.

**Figure 3:**
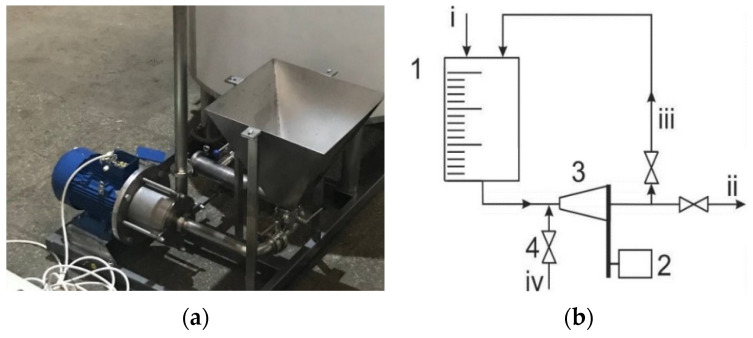
HSMD common view (**a**), homogeniser principal scheme (**b**), where: 1—peat-slurry tank; 2—electric motor; 3—HSMD; and 4—valve for extra component supply funnel (iv); i—water supply; ii—slurry output; and iii—recirculation flow.

The rotation speed of the HSMD used in the experiments was 8500–9000 min^−1^, and the linear velocity of the working teeth was from 70 to 80 m·s^−1^. Therefore, the cavitation conditions required for slurry homogenisation were ensured. The technological scheme and HSMD standard view are given in Figure 3. The treatment time by HSMD was 5 min, and 45 kg of the total amount of HP was used to ensure a homogenous sludge-like HP.

**Figure 4:**
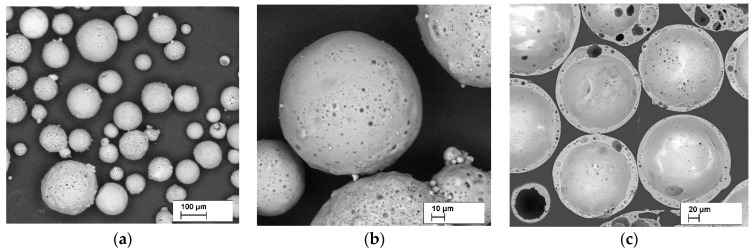
Scanning-electron-microscopy images of CS typical appearance at ×100 times magnification (**a**), at ×500 (**b**) and cross-section ×200 times magnification (**c**).

The CS used in the experiments were supplied by Biothecha Ltd. (Riga, Latvia). Chemical composition of the CS is as follows: SiO_2_—53.8 ± 0.5%; Al_2_O_3_—40.7 ± 0.7%; Fe_2_O_3_—1.0 ± 0.2%; CaO—1.4 ± 0.2%; MgO—0.6 ± 0.2%; Na_2_O—0.5 ± 0.1%; and K_2_O 0.4 ± 0.1%. Loss of ignition is 1.1 ± 0.1%. The grading composition is < 63 µm—1.70 wt.%, 63–75µm—3.86 wt.%, and 75–150—94.30 wt.%. CS average wall thickness is from 7 to 15 µm. A detailed characterisation, including chemical analysis, particle size and morphology, has been published in [2,3,29]. The common appearance of the CS is represented in Figure 4. 

**Figure 5:**
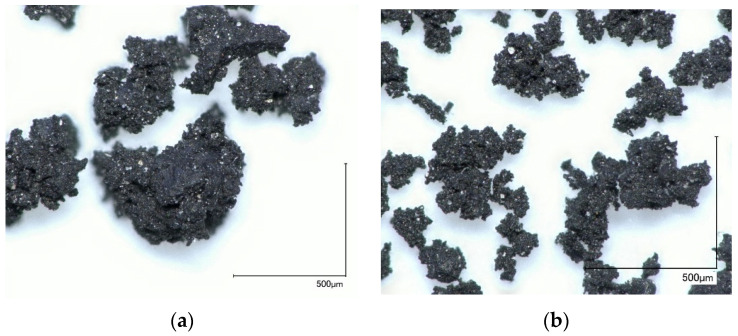
Digital optical micrographs of devulcanised crumb rubber (DCR) 0.25–0.5 mm (**a**) and <0.25 mm (**b**) size.

The DCR used for current research is provided by company Rubber Products Ltd. (Riga, Latvia). The DCR is produced using mechanochemical technology [30]. The manufacturing process comprises the processing crumb rubber by grinding at 60–70 °C with devulcanisation agent (urea) addition. The final product represents a sponge-like aggregate of DCR (average devulcanised rubber contents—13.4 wt%). For the DCR milling de-agglomeration, an impact-type disintegrator DESI-15 (Desintegraator Tootmise OÜ, Estonia) at a rotation speed of 3000 min^−1^ was used. The DCR was milled in direct mode five times (passes). For the present study, a 0.25–2.0 mm fraction was used (Figure 5). More details about DCR milling, particle size distribution, and morphology are described by Lapkovskis et al. [31].

For the production of the block, the components were manually mixed until homogeneous, then placed into plastic moulds of 140 × 180 × 20 mm^3^. Samples were dried at room temperature for 20 days. After drying, all specimens were demoulded and left for ambient drying for ten days. For removing any residual humidity, samples were dried at 105 °C for 48 h.

**Figure 6:**
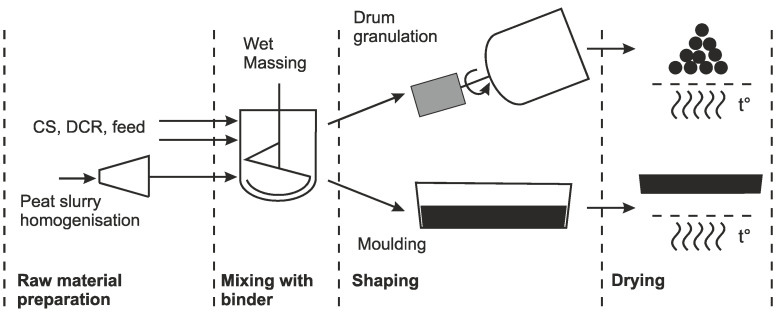
Principal scheme for producing the bio-based binder composite material in the shape of granules and blocks.

For the granules, the components were manually mixed until homogeneous, then placed in a rotary-drum granulator with a drum diameter of 950 mm and rotation speed of 80 s^−1^. Samples were dried at room temperature for 2 days. To remove any residual humidity, specimens were dried at 105 °C for 48 h. The standard production scheme of composite blocks and granules is illustrated in Figure 6. 

### 2.2. Characterisation Methods

#### Liquid Adsorption

Determination of liquid (water and oil products) absorption was performed by immersing specimens in the liquid and checking the weight at a specific interval. The experiments were repeated five times for each composition/liquid, with a margin of error relative to the mean for each experiment. The liquid absorption (W) is calculated according to Equation (1):

(1) $$W=\frac{m_{1}-m_{0}}{m_{0}}$$

where

*m*_1_—the mass of the sample saturated with liquid, g; 

*m*_0_—dry mass (before immersion) of the sample, g; and

*W*—liquid absorption g/g.

### 2.3. Used Equipment and Measurement Devices

A high-speed multidisc mixer–disperser with cavitation effect (HSMD) [32,33,34] was used for obtaining a homogeneous water–peat slurry with a dry-matter content of 15 ± 1 wt.%. The moisture content was determined using a moisture analyser Kern MRS 120-3. Measurements were repeated seven times using the standard deviation to determine the standard error from the arithmetic mean. The Clatronic Multi Food Processor KM3350 (Clatronic GmbH, Kempen, Germany) with stainless steel container and a rubber-coated anchor-type mixer was used for the wet-mixture preparation at a rotation speed of 60 min^−1.^

For examining the specimens, a micro-optical inspection digital light microscope Keyence VHX-1000 (Keyence Corp. Osaka, Japan) equipped with digital camera 54MPx and VH-112 Z20R/Z20W lens, scanning electron microscopy (SEM)—field emission SEM Tescan Mira/LMU (Dortmund, Germany)*,* and optical microscopy were used.

## 3. Results

### 3.1. Morphology of the Obtained Biocomposite Block and Granules

**Figure 7:**
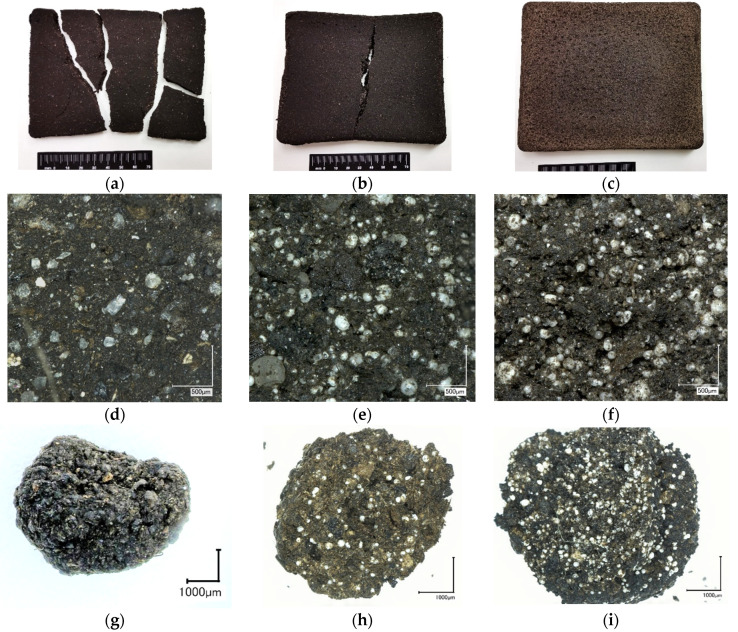
Images of CS–DCR–homogenised peat (HP) composite material: common appearance of dried block 0–100–0 (**a**), 5–90–5 (**b**), and 20–70–10 (**c**); micro-images of blocks 0–100–0 (**d**), 5–90–5 (**e**), 20–70–10 (**f**) fractures: and granules: 0–100–0 (**g**), 5–90–5 (**h**), and 20–70–10 (**i**) common appearances.

The most characteristic differences of the obtained biocomposites morphology in the form of block and granules are shown in Figure 7. The most significant difference in the appearance of the obtained composites is noted for the block-shaped material with 0, 5, and 10 wt.% of CS. The specimens containing 100 wt.% of HP (composition 0–100–0) were intensely cracked after drying (Figure 7a), demonstrating a high shrinkage. This is attributed to the used HP without any additive containing 85 wt.% of water. Detailed visual inspection of the cracked specimen’s parts, using magnification X50 times (Figure 7d) shows a dense non-porous structure with white, crystal-like inclusions—sand particles. After analysis in polarised light, mainly quartz particles and an admixture of limestone were discovered, these being a natural component of the Baltic-region peat. The addition of 5 wt.% of CS and/or 5 wt.% of DCR strongly minimised the shrinkage and cracking. The typical appearance of the 0–95–5, 5–95–0, and 5–90–5 specimens is illustrated in Figure 7b. However, in comparison with highly-loaded composition 20–70–10, its geometry differs from mould shape (Figure 7b,c). Nevertheless, it is necessary to consider that the real content of fillers CS and DCR is much higher (Table 1, Table 2) because the water loss from HP increases the CS and CDR content in the composite. Specimens 0–95–5, 5–95–0, and 5–95–5 after drying have 0–72.7–27.3, 27.3–72.7–0, and 22.1–55.8–22.1 CDR–HP–CR mass ratio (or weight %), respectively. The shrinkage-ratio decrease has been reported by several works [2,35,36], mainly with a ceramic matrix material where a high shrinkage is usually observed during the drying and firing [2,37].

In contrasts with the block material, the 0-100-0 granules have no significant morphological differences with the other composition specimens (Figure 7g–i). All the manufactured granules are characterised by a near-spherical shape and the particle-size distribution for all composition was: 1–2 mm—7–15%, 2–6 mm—10–20, and 6–10 mm—60–70 wt.%. 

### 3.2. Mechanical Properties and Density of the Obtained Biocomposite Block and Granules

**Figure 8:**
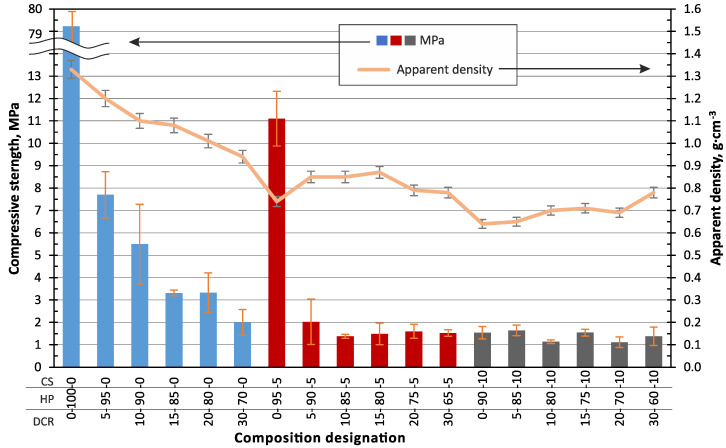
Dependence of the apparent density and compressive strength of the biocomposite in the shape of a block on the DCR and CS fraction. The composition of DCR HP and CS fraction is indicated by weight % in the wet mixture.

The obtained composites in the form of blocks were tested for compression strength and apparent density. The results are represented in a combined diagram in Figure 8. It can be seen that the highest compression strength of 79 MPa corresponds to the pure peat-based bio-binder (0–100–0). The second-highest compression strength of 11 MPa corresponds to the 0–100–5 composition with 5 wt.% of CS in the raw wet mixture or 27.3 wt.% in the composite material after drying (Table 1). The observation of the parts of the cracked specimens 0–100–5 (with 27.3 wt.% of CS) revealed a dense structure without cracks or voids, the same as 0–100–0 (100 wt.% of HP, Figure 7d) specimens. A significant difference in mechanical properties (79.3 and 11.1 MPa) could be explained by the presence of the filler with lower mechanical properties than the quartz and limestone particles of the CS. The introduction of 27.3 wt.% of the DCR leads to a decrease in the compression strength to 7.6 MPa.

In all the studied cases, an increase of the CDR in the composites leads to a significant decrease of compression strength, up to 1.5 ± 0.4 MPa, but not less than 1.1 MPa (10–80–10 and 20–70–10). 

**Figure 9:**
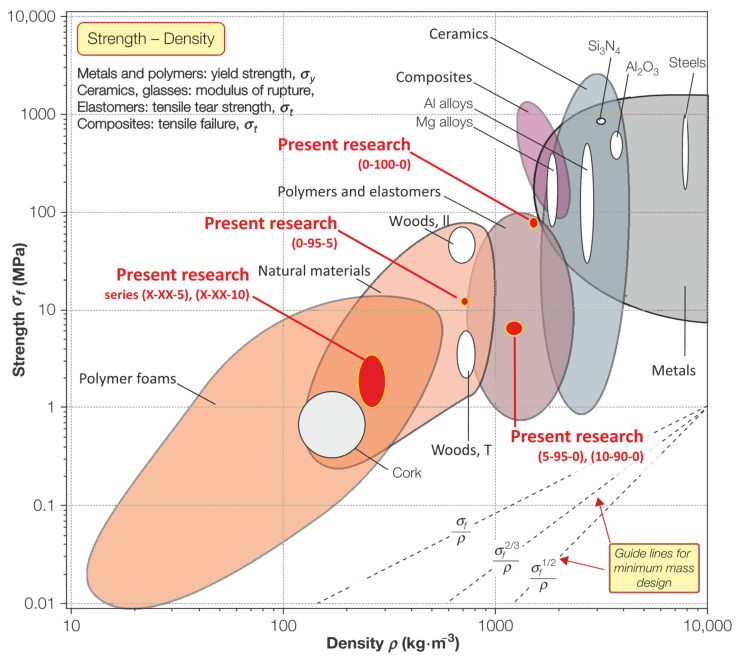
Influence on the compliance of studied biocomposites (in the form of a block) with typical materials demonstrated in the Ashby classification diagram (adapted from [38]).

By applying the determined physical–mechanical properties data of the obtained samples to Ashby’s [38] compression strength and density summary diagram (Figure 9), it can be concluded that the obtained material demonstrates a relatively low density and relatively high strength, characteristic of biocomposites, which is one of the key aims of this work. Pure bio-binder (0–100–0) composite material in units MPa—kg·m^−3^, is characterised by such property combinations that it is located near to the three different types of materials (metals, ceramics, and polymers), which is a unique properties combination and much materials belong to such property’s combinations. Compositions 5–XX–XX and 10–XX–XX with 5 and 10 wt.% of DCR content in wet mixture and units MPa—kg·m^−3^, belong to the lower zone of the natural material area. 

### 3.3. Sorption of Liquids in the Structure of the Granulated Biocomposites

**Figure 10:**
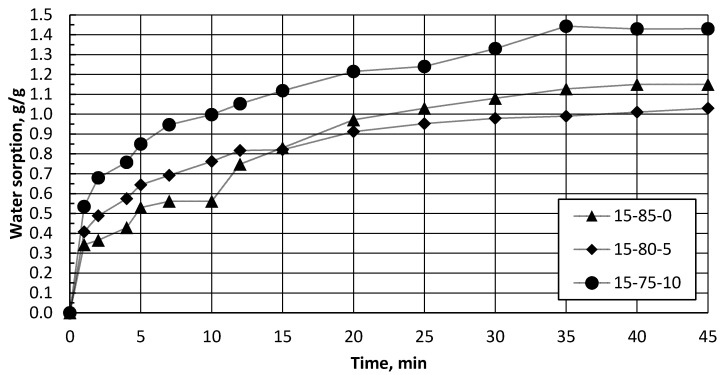
Water adsorption, in g/g for granules compositions series with 0 wt.% (15–85–0), 5 wt.% (15–80–5), and 10 wt.% (15–75–10) of CS.

**Figure 11:**
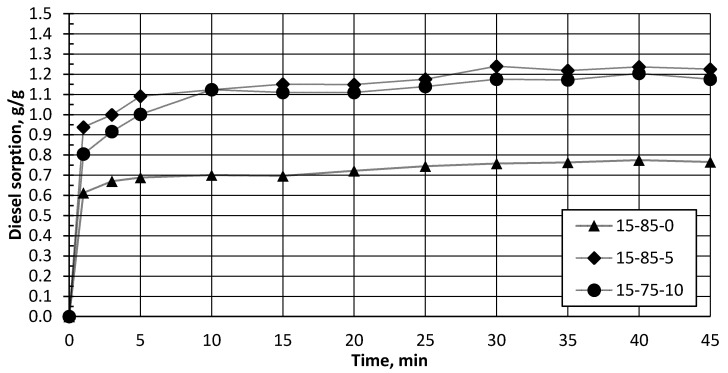
Diesel adsorption, in g/g for granules compositions series with 0 wt.% (15–85–0), 5 wt.% (15–85–5), and 10 wt.% (15–75–10) of CS.

The obtained biocomposite granules were used for sorption of water and oil products (diesel). Sorption kinetics were estimated for the developed biocomposite using diesel fuel as a model compound, as demonstrated in Figure 10 and Figure 11. All samples reached a 90% water-sorbent uptake capacity in 25–30 min, with maximal saturation after 35–45 min Figure 10. All the samples’ series demonstrated a near 1.0 g·g^−1^ water-sorption-capacity saturation. A 90% sorbent uptake capacity was noted for the diesel in a shorter time, in 5–10 min, with a maximal saturation after 35–45 min Figure 11. The samples’ series demonstrated from 1.0 to 1.5 g·g^−1^ diesel sorption capacity at equilibrium conditions. The highest adsorption capacity was 1.5 g·g^−1^ for specimen 30–65–5, which corresponds to a 68.0–20.6–11.3 ratio of the dry composite components. It is necessary to admit that liquid’s maximal saturation was for diesel, with maximal saturation reached within 3–5 min. 

**Figure 12:**
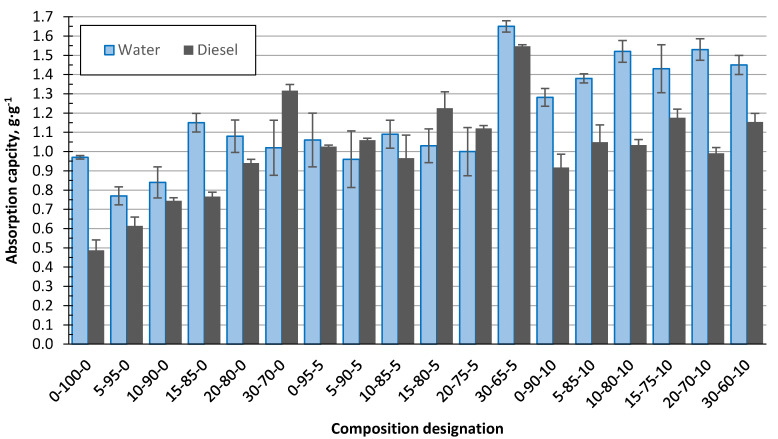
Sorbent water and diesel uptake capacity in g/g for granules compositions.

Figure 12 illustrates the water and diesel uptake capacity, in g/g, for granules, and it can be seen that for most cases (except 30–70–0, 5–90–5, 15–80–5, and 20–75–5), there is greater sorption for water. For the composition series XX–XX–0 and XX–XX–10, the water uptake is significantly higher than for diesel, from 10 to 50%, but for the XX–XX–5 series, there is no significant difference between the water and diesel uptake. However, considering the sorption-capacity ratio from the mass ratio [g·g^−1^] of the sorbent mass to the absorbed-liquid volume [cm^3^·g], the sorbent capacity for diesel is higher by 15%. The diesel density was assumed as 0.85 g·cm^−3^. 

## 4. Conclusions

In the current research, a three-phase composite material containing homogenised peat as a bio-binder for water and oil products was produced in the form of blocks and granules for the first time. The obtained material in the form of a block was characterised by the right combination of compressive strength and density.

The obtained granulated sorbent containing 68.0–20.6–11.3 of CDR HP and CS demonstrated up to 1.5 g·g^−1^ maximal sorption capacity for diesel.

The composite material with CS content of 27.3 wt.% is characterised by the highest value (except for the pure bio-binder) of compression strength of 11.2 MPa and at the same time an apparent density of 0.75 g·cm^−3^. HP as a bio-binder and CS as a lightweight filler could become a prospective material for designing lightweight bio-based structures. Further investigations of CS content’s influence on the CS–HP biocomposite are foreseen and usage for acoustic and thermal insulation to be explored.

## Figures and Tables

**Table 1:** The composition of block and granules in a raw mixture (wet) and after drying, by wt.% (part I).

	**Designation of the Composition**											
	0–100–0	5–95–0	10–90–0	15–85–0	20–80–0	30–70–0	0–95–5	5–90–5	10–85–5	15–80–5	20–75–5	30–65–5
**Wet mixture composition [wt.%]**						
DCR	0.0	5.0	10.0	15.0	20.0	30.0	0.0	5.0	10.0	15.0	20.0	30.0
HP	100	95.0	90.0	85.0	80.0	70.0	95.0	90.0	85.0	80.0	75.0	65.0
CS	0.0	0.0	0.0	0.0	0.0	0.0	5.0	5.0	5.0	5.0	5.0	5.0
	**Dried composite material formulation [wt.%]**											
DCR	0.0	27.3	44.2	55.8	64.1	75.4	0.0	22.1	37.2	48.1	56.3	68.0
HP	100	72.7	55.8	44.2	35.9	24.6	72.7	55.8	44.2	35.9	29.6	20.6
CS	0.0	0.0	0.0	0.0	0.0	0.0	27.3	22.1	18.6	16.0	14.1	11.3

**Table 2:** The composition of block and granules in a raw mixture (wet) and after drying, by wt.% (part II).

**Designation of the Composition**						
	0–90–10	5–85–10	10–80–10	15–75–10	20–70–10	30–60–10
**Wet mixture composition [wt.%]**						
DCR	0.0	5.0	10.0	15.0	20.0	30.0
HP	90.0	85.0	80.0	75.0	70.0	60.0
CS	10.0	10.0	10.0	10.0	10.0	10.0
**Dried composite material formulation [wt.%]**						
DCR	0.0	18.6	32.1	42.3	50.3	62.0
HP	55.8	44.2	35.9	29.6	24.6	17.4
CS	44.2	37.2	32.1	28.2	25.1	20.7
